# Causal relationship between oral diseases and hypertension: a Mendelian randomization study

**DOI:** 10.3389/ebm.2026.10922

**Published:** 2026-05-07

**Authors:** Bofan Qian, Zhiwen Fang

**Affiliations:** 1 Shanghai Engineering Research Center of Tooth Restoration and Regeneration and Tongji Research Institute of Stomatology, Shanghai Tongji Stomatological Hospital and Dental School, Tongji University, Shanghai, China; 2 Department of Statistics and Data Science, Tsinghua University, Beijing, China

**Keywords:** causal inference, essential hypertension, mendelian randomization, oral diseases, secondary hypertension

## Abstract

Current evidence supports the potential association between several common oral diseases and hypertension. The aim of the research is to clarify the causal relationship between these oral diseases and hypertension using Mendelian randomization (MR) analysis. Single nucleotide polymorphisms (SNPs) related to five oral traits (periodontitis, bleeding gums, loose teeth, periapical abscess and dental caries) were obtained from GWAS catalog, while those associated with hypertension (essential and secondary) were extracted from the FinnGen database. The SNPs were employed as instrumental variables (IVs) in the MR analysis. Assorted methods were applied, and inverse variance-weighted (IVW) analytical method was prioritized. Sensitivity analyses including MR-PRESSO method, MR Egger intercept test, Cochran’s Q test, leave-one-out analysis and MR Steiger test were conducted. Our analysis identified the potential causal relationship between dental caries and essential hypertension. The forward MR analysis demonstrated a significant causal effect of dental caries on essential hypertension (OR = 1.036, 95%CI: 1.012–1.059, *P* = 0.003). The reverse analysis also indicated a significant causal effect (OR = 1.160, 95%CI: 1.016–1.323, *P* = 0.028). Additionally, we observed a causal effect of bleeding gums on essential hypertension (OR = 1.145, 95%CI: 1.019–1.288, *P* = 0.023). These findings support the potential causality between specific oral diseases and essential hypertension.

## Impact statement

This paper addresses the issue of causal relationship between oral diseases and hypertension. Previous studies have shown that assorted oral diseases are possibly associated with hypertension. However, the results of these studies are biased. In addition, most of the studies are observational, which fail to reveal the causality and may be significantly affected by confounding factors and reverse causal relationship. In our study, we utilize Mendelian randomization analysis to investigate the causality between several oral diseases and hypertension, which minimizes the potential effect of confounders and reverse causality. Our study suggests that there is a bidirectional causal relationship between dental caries and essential hypertension, while gingival bleeding has a causal effect on essential hypertension. Our findings are significant as it can offer new insights into the association of the diseases, emphasizing the importance of oral health in the management of hypertension.

## Introduction

Oral diseases are prevalent but neglected globally [1]. It is estimated that nearly half of the world’s population suffers from oral diseases [2]. Among these diseases, dental caries and periodontal diseases are considered critical [3].

Hypertension is a common chronic disease which can be classified as essential or secondary [4]. In the cases of hypertension, essential hypertension accounts for the vast majority [5]. Several risk factors for essential hypertension have been identified, including diabetes, dyslipidemia, smoking and alcohol consumption [6]. However, the etiology of essential hypertension remains unclear.

In the previous research, the association between oral diseases and hypertension was described. Numerous studies have investigated the potential association between periodontitis and hypertension, and the results were polarized [7–11]. Evidence supports that severe periodontitis is associated with hypertension [12]. Gingival bleeding, as a surrogate for active periodontal inflammation, has been found to be positively correlated with hypertension [13]. In addition, loose teeth can also to some extent reflect the severity of periodontitis [14]. Another cross-sectional study has indicated that periapical abscess is closely associated with both essential and secondary hypertension, however, controversy still exists [15, 16]. Dental caries, with an extremely high prevalence, has also been linked to essential hypertension [17–19]. However, most of the studies are observational, indicating that confounding factors still exist and the direction of the relationships cannot be ascertained.

Mendelian randomization (MR) is an emerging innovative analytical approach similar to randomized controlled trials (RCTs), which uses genetic variance as instrumental variables (IVs), assessing the influence of risk factors on outcomes. MR analysis can overcome unmeasured confounding factors which may lead to deviation in traditional observational studies [20]. Additionally, it can effectively identify the direction of causal relationships and reduce the impact of reverse causality to a large extent [21]. Therefore, this study aims to utilize data extracted from GWAS catalog and FinnGen database to conduct two-sample MR analyses, offering insights into the correlation between several oral diseases and hypertension.

## Materials and methods

### Study design

**FIGURE 1:**
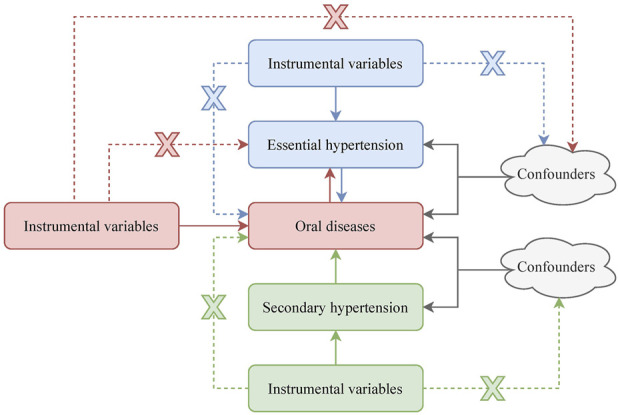
Study design of Mendelian randomization analysis.

This study was in full compliance with the STROBE-MR statement [22]. To conduct the MR analysis, three assumptions ought to be satisfied: (1) Selected IVs should be significantly related to the exposure; (2) Selected IVs must be independent of the confounding factors; (3) Selected IVs can only influence the outcome by exposure. The design of our study is depicted in Figure 1. First, a bidirectional MR analysis was implemented to investigate potential causal relationships between oral diseases and essential hypertension. Then, a unidirectional MR analysis was conducted to examine the effect of secondary hypertension on oral diseases.

### Data sources

Data used in the research were extracted from two different genome-wide association study (GWAS) databases, effectively avoiding sample overlapping [23]. SNPs related to oral health conditions and hypertension were extracted from the GWAS catalog website^1^ and the FinnGen website^2^ respectively. The relevant features of samples are displayed in Table 1.

**TABLE 1:** Relevant features of samples used in MR analysis.

Trait	Consortium	GWAS ID	Population	Samplesize	N case
Periodontitis	GWAS catalog	GCST90436269 [24]	European	399358	1222
Bleeding gums	GWAS catalog	GCST90044343 [25]	European	454565	59211
Loose teeth	GWAS catalog	GCST90044344 [25]	European	454565	18545
Periapical abscess	GWAS catalog	GCST90436266 [24]	European	399313	1177
Dental caries	GWAS catalog	GCST90044098 [25]	European	456348	2906
Essential hypertension	FinnGen	finngen_R12_I9_HYPTENSESS [26]	European	478149	132515
Secondary hypertension	FinnGen	finngen_R12_I9_HYPTENSEC [26]	European	256198	3388

### Selection of instrumental variables

Regarding instrumental variables for essential and secondary hypertension, the *P* value was set at 5 × 10^−8^. Due to the insufficient number of SNPs for oral traits, we relaxed the threshold to 5 × 10^−7^ for bleeding gums and 5 × 10^−6^ for other traits [27–29]. Linkage disequilibrium (LD) was performed using standard clumping parameters (*r* ^2^ < 0.001, clumping window = 10,000 kb). Confounders were identified based on previous epidemiological and genetic studies, including well-established common risk factors for essential hypertension (e.g., body mass index, metabolite levels, and hemoglobin concentration) [30–32]. Subsequently, we imported the selected SNPs into the LDtrait tool^3^ to assess their associations with the confounders, and any SNP exhibiting a significant association with any of these confounders was excluded [33]. To estimate the strength of the IVs, we calculated *F*-statistic in turn utilizing the following formula:

$$F={\left(\frac{\beta }{SE}\right)}^{2}.$$

Normally, an *F*-statistic >10 implies no obvious bias generated by weak instrumental variable [34]. Considering the excessive quantity of SNPs for essential hypertension, we adopted a relatively strict *F*-statistic (*F* > 50). Finally, we acquired the IVs used for the subsequent MR analysis (Supplementary Material 1).

### Mendelian randomization analysis

All the procedures were completed in R software (version 4.4.2), utilizing “TwoSampleMR” (version 0.6.8) and “MR-PRESSO” (version 1.0) R packages. First, records of exposure and outcome were merged, and missing SNPs were deleted. Then, duplicated and palindromic SNPs were excluded. The MR-PRESSO approach was employed to detect potential horizontal pleiotropy [35]. After manually excluding the outliers outputted by MR-PRESSO, the process was repeated until no outlier was identified. The SNPs undergoing these processes can be used in the subsequent analysis. In our analysis, inverse variance weighting (IVW) served as the main analytical approach [36]. IVW estimates the weighted average of Wald ratios for the SNPs, assuming the validity of each SNP, and provides the strongest statistical power. The IVW random-effect model was employed because it is robust even in the presence of heterogeneity. Additionally, MR Egger and weighted median methods served as complementary methods. The former method is based on the assumption of instrument strength independent of direct effect (InSIDE), which can detect horizontal pleiotropy via the intercept term [37]. When the intercept term is not significantly different from zero, this suggests no evidence of horizontal pleiotropy, and the results are consistent with those from the IVW method. The weighted median method can provide a consistent causal estimate even when up to 50% of the IVs are invalid [38]. Collectively, these methods were used to evaluate the stability of the causal estimates. Consistent results across different approaches would strengthen the credibility of the causal association, whereas divergent results may indicate the presence of potential bias such as horizontal pleiotropy. The MR Egger intercept test was conducted to validate horizontal pleiotropy. In our test, a *P* < 0.05 indicates that horizontal pleiotropy existed in the results. In addition, Cochran’s Q test was conducted to assess heterogeneity, and a *P* < 0.05 indicates the existence of heterogeneity. Leave-one-out sensitivity analysis was performed to assess the stability of the causal estimates. Although outliers had already been excluded by MR-PRESSO, the leave-one-out test verified that no single SNP predominantly influenced the overall effect, further supporting the robustness of our findings [39]. Furthermore, we conducted MR Steiger test to determine the direction of the causality by comparing the strength of association between IVs and the exposure versus the outcome [40]. In the Steiger test, a *P* < 0.05 supports the direction of causal relationship from exposure to outcome, with no evidence of reverse causality.

## Results

Forest plots, scatter plots, funnel plots and the plot of leave-one-out sensitivity analysis were formed in R software (Supplementary Material 2). The results of Steiger test were shown in Supplementary Material 3. No heterogeneity or pleiotropy was discovered in the MR analysis (Table 2).

**TABLE 2:** The results of heterogeneity and pleiotropy.

Exposure	Outcome	*P*_Cochran’s Q_	*P*_MR Egger intercept_	*P*_Global test_
Periodontitis	Essential hypertension	0.284	0.280	0.322
Bleeding gums	​	0.142	0.734	0.181
Loose teeth	​	0.179	0.802	0.199
Periapical abscess	​	0.095	0.597	0.135
Dental caries	​	0.444	0.217	0.478
Essential hypertension	Periodontitis	0.266	0.479	0.230
​	Bleeding gums	0.081	0.161	0.072
​	Loose teeth	0.076	0.952	0.066
​	Periapical abscess	0.261	0.459	0.228
​	Dental caries	0.264	0.482	0.277
Secondary hypertension	Periodontitis	0.472	0.928	0.478
​	Bleeding gums	0.605	0.686	0.392
​	Loose teeth	0.127	0.405	0.200
​	Periapical abscess	0.441	0.630	0.451
​	Dental caries	0.573	0.384	0.492

### Causal effects of oral traits on essential hypertension

**FIGURE 2:**
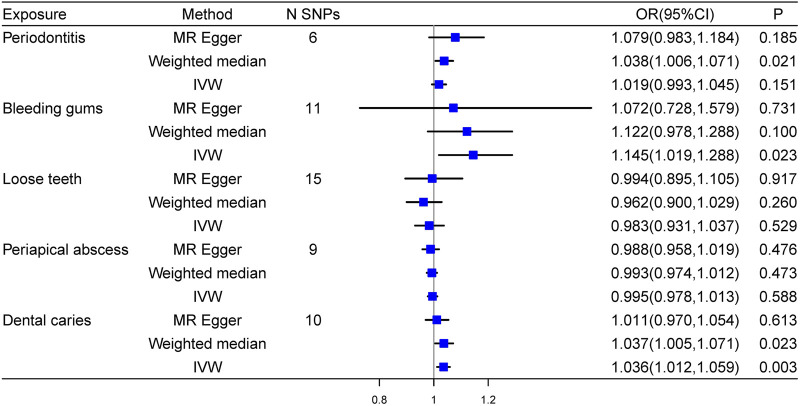
Forest plot of the causal effects of oral traits on essential hypertension.

Our research revealed that bleeding gums (OR = 1.145, 95%CI: 1.019–1.288, *P* = 0.023) and dental caries (OR = 1.036, 95%CI: 1.012–1.059, *P* = 0.003) were positively correlated with essential hypertension. Although the weighted median analysis indicated the correlation between periodontitis and essential hypertension (OR = 1.038, 95%CI: 1.006–1.071, *P* = 0.021), it didn’t serve as the major analytical approach. Therefore, no causal effects of other three oral traits on essential hypertension were identified. Detailed results were shown in Figure 2.

### Causal effects of essential hypertension on oral traits

**FIGURE 3:**
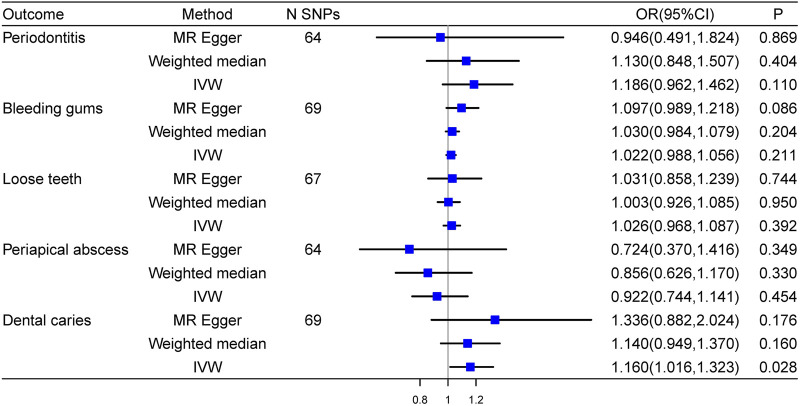
Forest plot of the causal effects of essential hypertension on oral traits.

After reversing the exposure and outcome, we discovered the causal effect of essential hypertension on dental caries (OR = 1.160, 95%CI: 1.016–1.323, *P* = 0.028), which indicated that the causality between dental caries and essential hypertension was bidirectional. Detailed results were shown in Figure 3.

### Causal effects of secondary hypertension on oral traits

**FIGURE 4:**
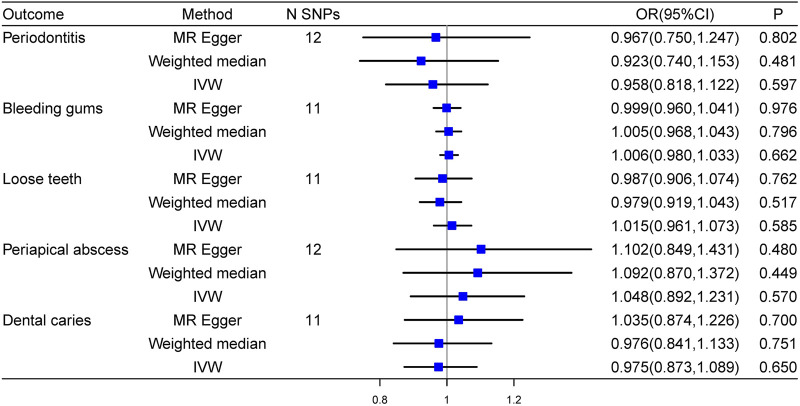
Forest plot of the causal effects of secondary hypertension on oral traits.

We also conducted a unidirectional MR analysis to evaluate the causal effect of secondary hypertension on oral diseases. No causal effect of secondary hypertension on oral traits was identified in the analysis. Detailed results were shown in Figure 4.

## Discussion

Oral diseases have already been found to be potentially associated with assorted systemic diseases in previous MR studies. Examples include kidney cancer, hypothyroidism and psychiatric disorders [41–43]. However, the majority of hypertension-related studies have placed emphasis on periodontitis [44, 45]. In fact, the oral health should be regarded as a whole. In our research, a comprehensive MR analysis was implemented to identify the causal relationship between several critical oral diseases and hypertension, eliminating the potential effect of confounders and reverse causal relationship to a great extent compared with observational studies. We stratified hypertension into two subtypes and examined the causality between oral diseases and each subtype separately. The results demonstrated the bidirectional causal relationship between dental caries and essential hypertension, and bleeding gums exerted a causal effect on essential hypertension.

According to previous research, chronic inflammatory process possibly participates in the pathogenesis of hypertension [46, 47]. Periodontitis, defined as a chronic inflammation affecting the periodontal tissues, can result in systemic inflammation and elevate the levels of inflammatory mediators including interleukin-6 (IL-6) and C-reactive protein (CRP), which may serve as mediators underlying the association [3, 48–50]. Although numerous studies have suggested a potential association between periodontitis and hypertension, our result contradicts these studies. Loose teeth, as an indicator, was also not associated with hypertension in our research. Interestingly, the causal effect of bleeding gums on essential hypertension was identified. This is consistent with a recent study, and a plausible explanation is that gingival bleeding represents a status of active periodontal inflammation, which is more likely to be associated with systemic inflammation compared with stable periodontitis, indicating that active periodontal inflammation might increase the risk of essential hypertension [12, 13].

Different from periodontitis, periapical diseases are caused by the infection of root canal system with no apparent symptoms [51]. Recent evidence supports the potential association between periapical abscess and hypertension, but the views are controversial. A large-scale cross-sectional study has found that patients with secondary hypertension are more likely to suffer from periapical abscess than those with essential hypertension [15]. However, our MR analysis doesn’t reveal such a relationship.

Dental caries, as a common oral disease both exist in primary and permament teeth, is also significantly correlated with essential hypertension [18, 19]. Surprisingly, our study supports this viewpoint to some extent and reveals the direction of the relationship. The result turns out to be bidirectional, but the sample used in our analysis is merely described as dental caries without detailed subgroups.

The influence of dental caries on vascular health is mostly attributed to the nature of this disease, which is primarily a chronic bacterial infection. It exerts local effects such as compromised tooth structure and periodontal support, as well as systemic effects by increasing circulating inflammatory mediators, which contribute to endothelial dysfunction and arterial plaque formation [19]. In addition, hypertensive patients with poor oral health status show intensified oxidation of several plasma substrates, increased reactive oxygen species (ROS), lipid peroxidation, inactivation of prostacyclin and NO, as well as an imbalance in total antioxidant capacity [19]. In contrast, the reverse effect can be attributed to xerostomia induced by hypertension [52]. Saliva performs multiple physiological functions in the oral cavity, in which its clearance effect prevents tooth demineralization and ensures microbial homeostasis [53]. Degeneration of the salivary glands in hypertensive patients leads to hyposalivation, increasing the risk of caries on different tooth surfaces [54].

Emphasis should be laid on oral health, with extra attention paid to patients suffering from common oral diseases, as dental caries and bleeding gums are closely associated with essential hypertension according to our research. A prospective cohort study has discovered that nonsurgical periodontal treatment can significantly reduce the level of blood pressure in patients with periodontitis and prehypertension, stressing the importance of oral health from another angle [55]. Besides, the number of teeth may affect the level of blood pressure, which is not involved in our study due to data limitations [56, 57]. Further study needs to be conducted on a larger scale and can be extended to the association between oral disease and cardiovascular diseases instead of merely hypertension [58].

Limitations exist in our study. Though restricting data to European populations can effectively avoid sample overlap, it limits the generalizability of the results. Additionally, relaxed SNP selection thresholds were adopted for several oral traits due to the limited availability of genome-wide significant variants. The use of relatively weaker instrumental variables may introduce potential bias and influence the precision of causal estimates to some extent [34]. Therefore, the findings from these traits should be interpreted with appropriate caution. Moreover, the lack of detailed subgroup data (e.g., primary and permanent dental caries) represents another limitation. Larger-scale GWAS are urgently needed to further investigate and validate these associations.

## Conclusion

In our research, we identified the bidirectional causal relationship between dental caries and essential hypertension, and also uncovered the causal effect of bleeding gums on essential hypertension. No causal associations were observed between other oral traits and essential hypertension. In addition, our results did not support the causal effect of secondary hypertension on oral diseases.

## Data Availability

The original contributions presented in the study are included in the article/Supplementary Material, further inquiries can be directed to the corresponding author.

## Footnotes

1
https://www.ebi.ac.uk/gwas/

2
https://r12.finngen.fi/

3
https://ldlink.nih.gov/?tab=ldtrait
