# Supplementary material for: Causal relationship between oral diseases and hypertension: a Mendelian randomization study
Source: Exp Biol Med (Maywood). 2026 May 7;251:10922. doi: 10.3389/ebm.2026.10922 (PMC13189983; doi:10.3389/ebm.2026.10922)
Supplement: Supplementary file 1 [file DataSheet2.pdf]

Summarized plots

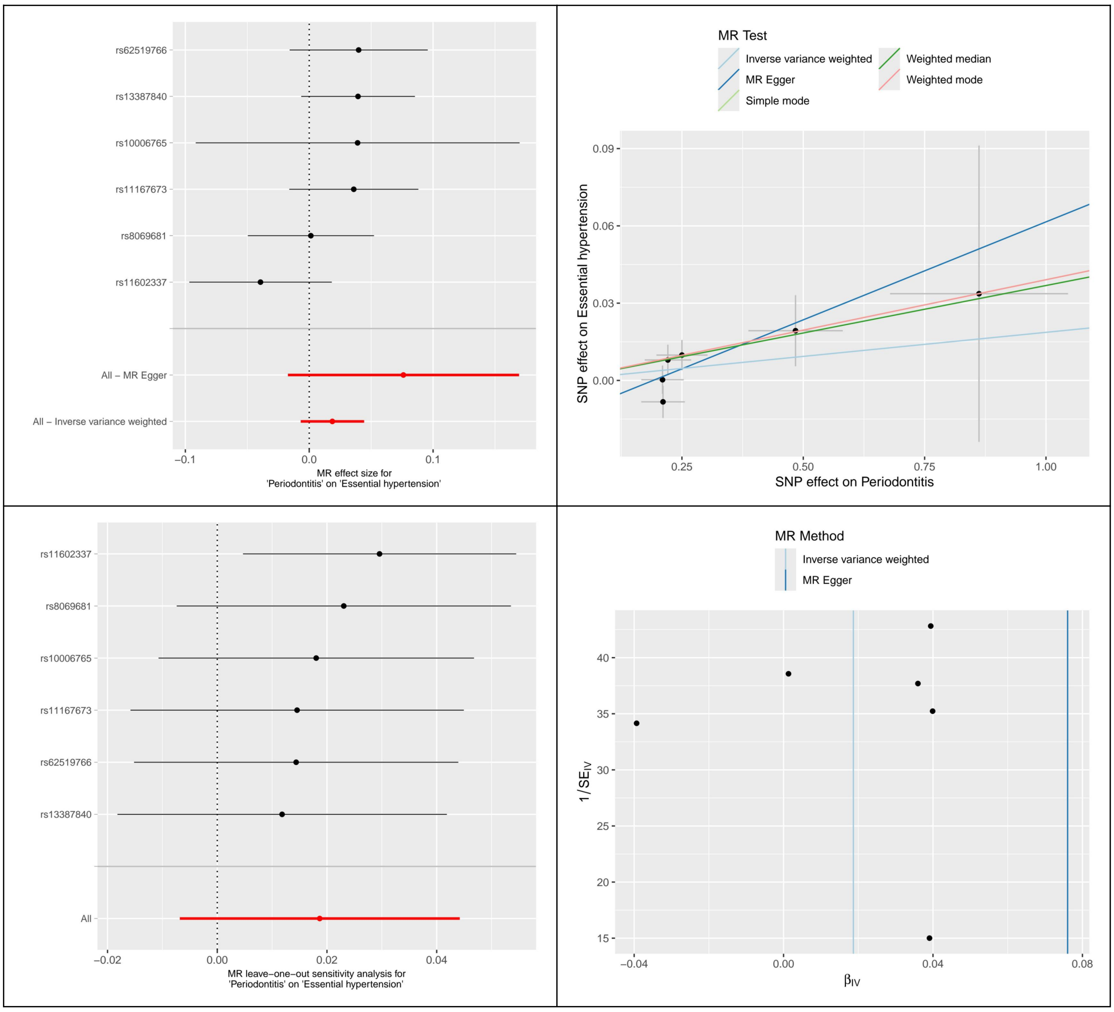

Periodontitis on Essential hypertension

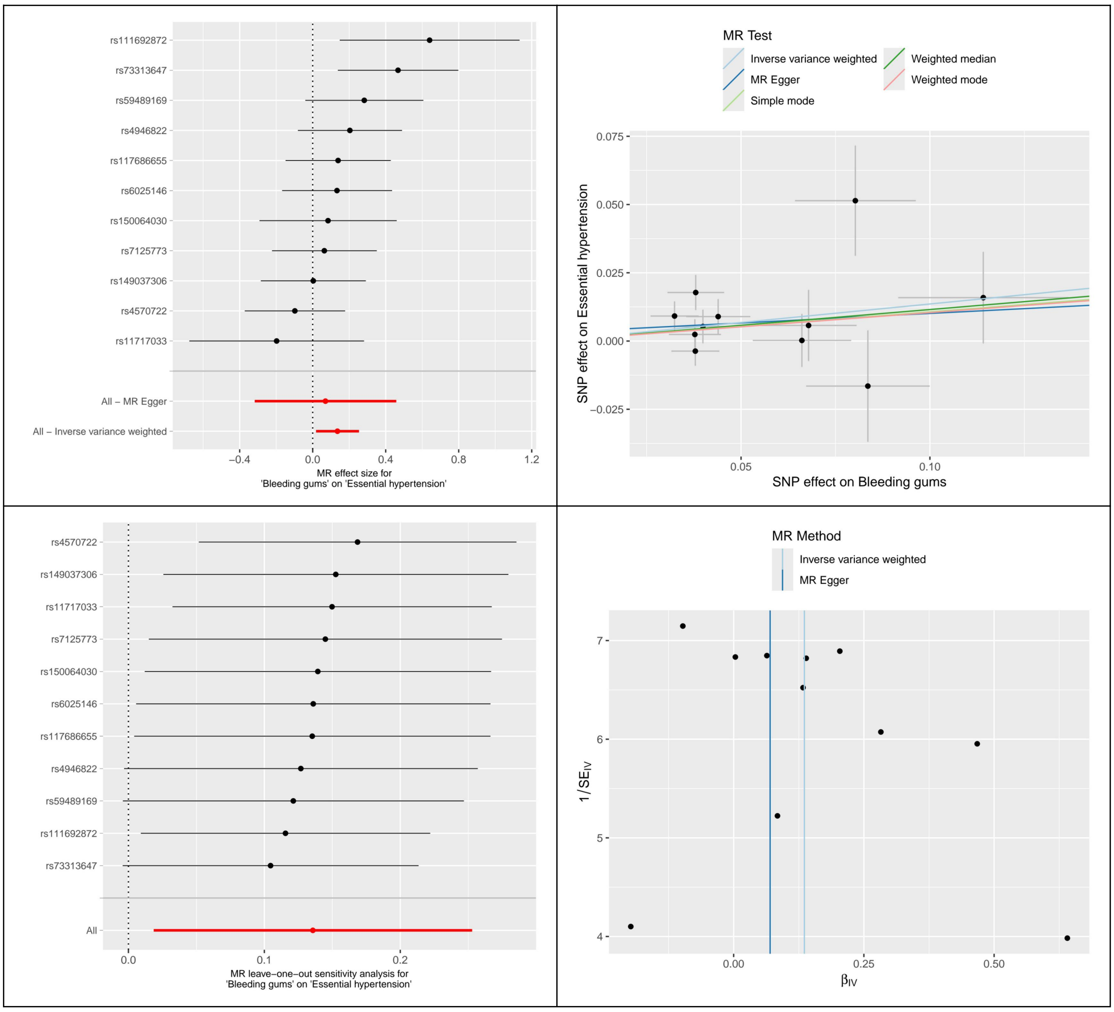

Bleeding gums on Essential hypertension

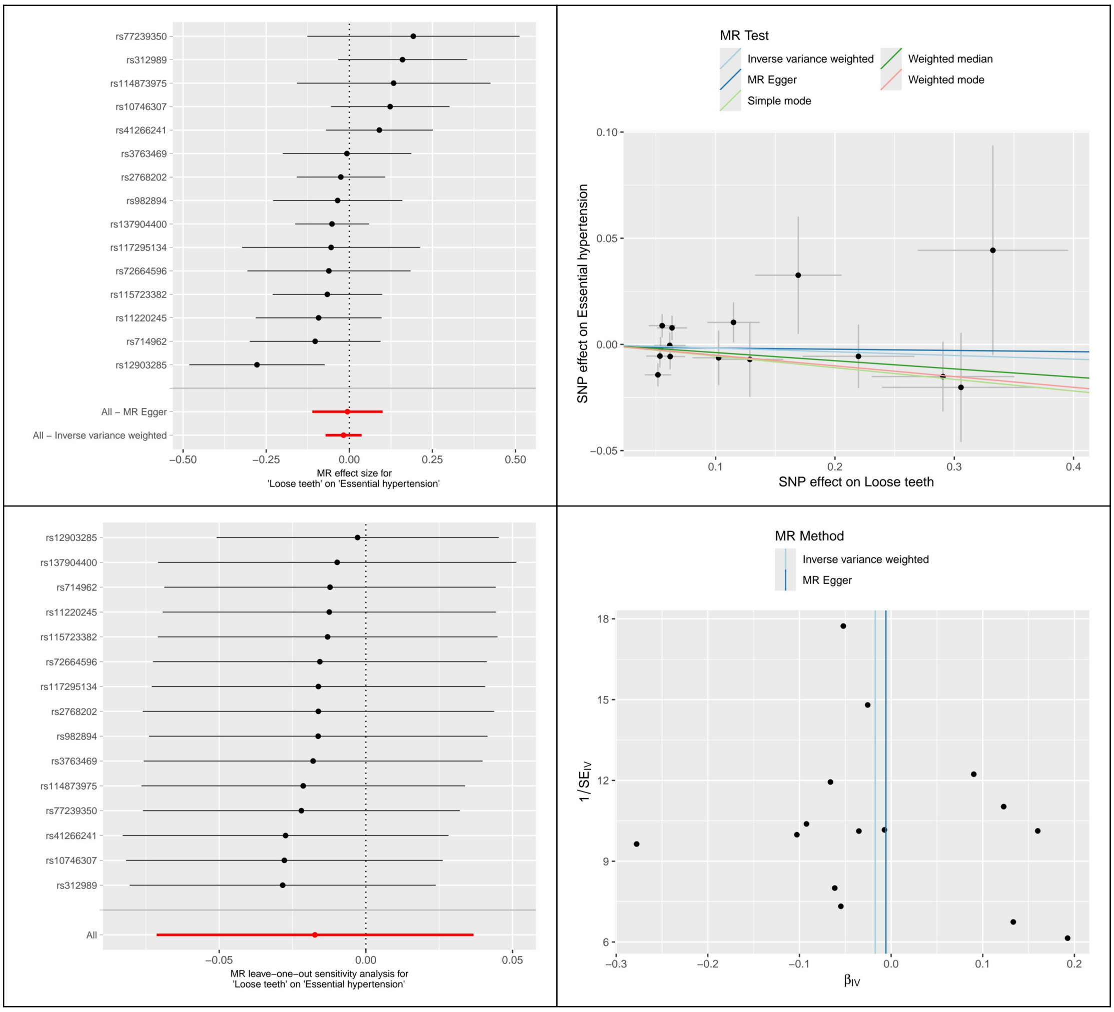

Loose teeth on Essential hypertension

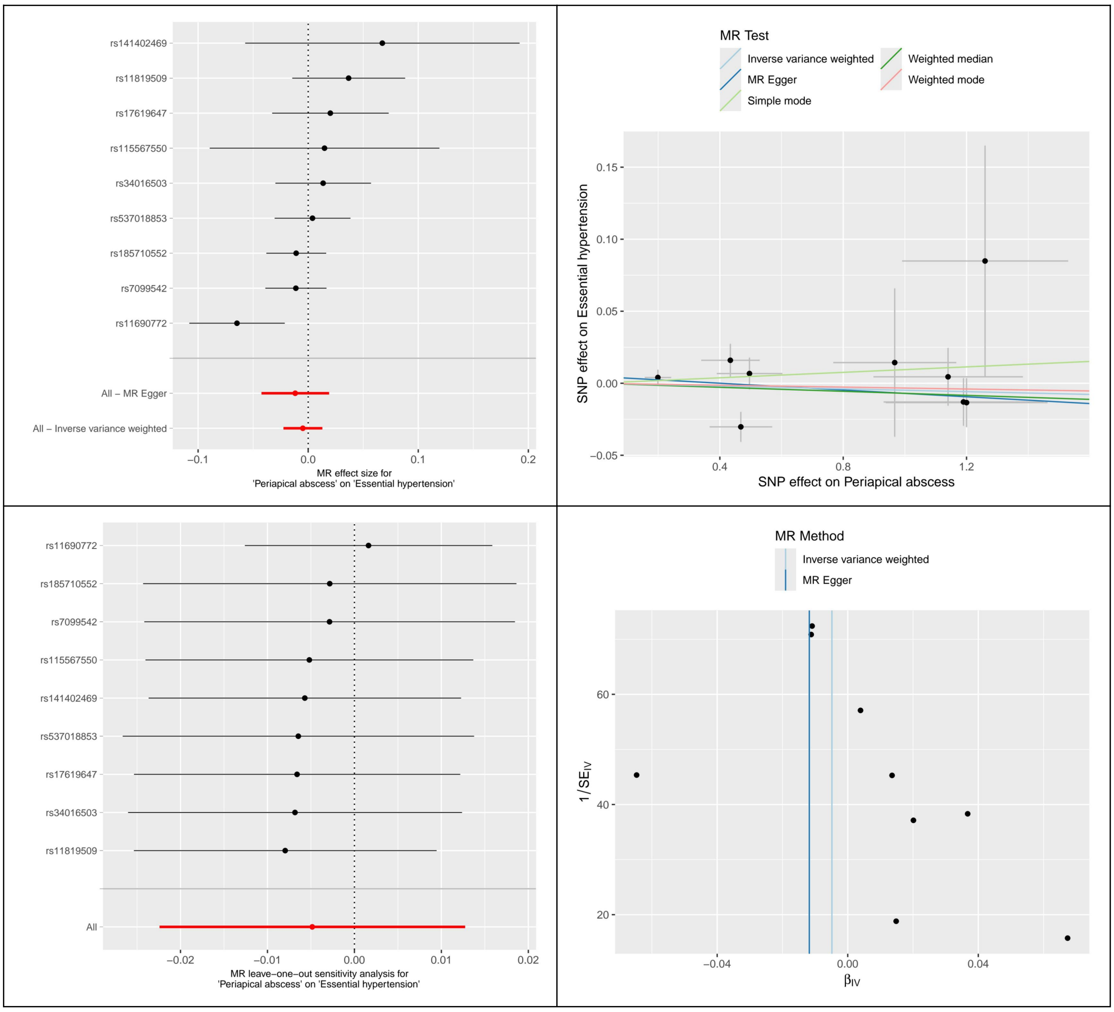

Periapical abscess on Essential hypertension

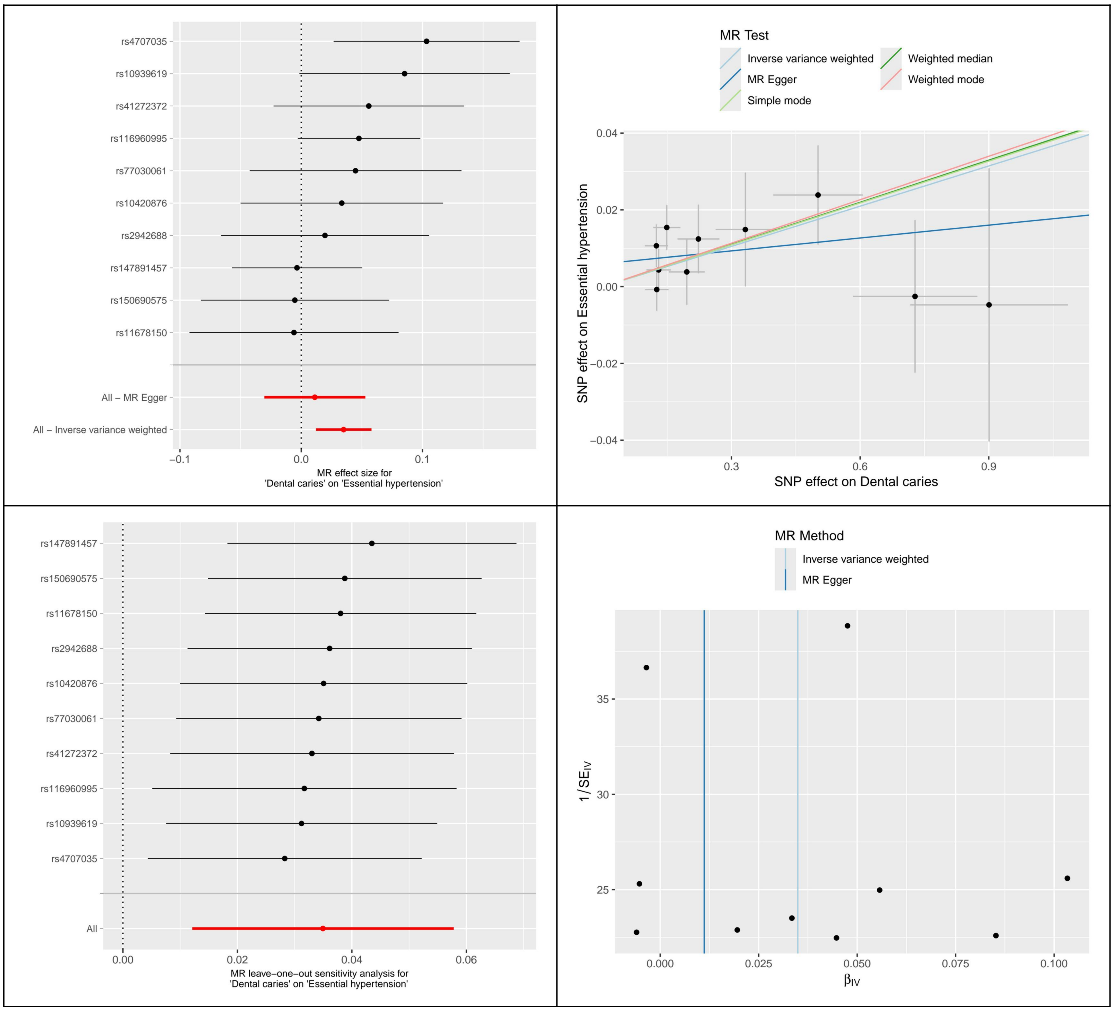

Dental caries on Essential hypertension

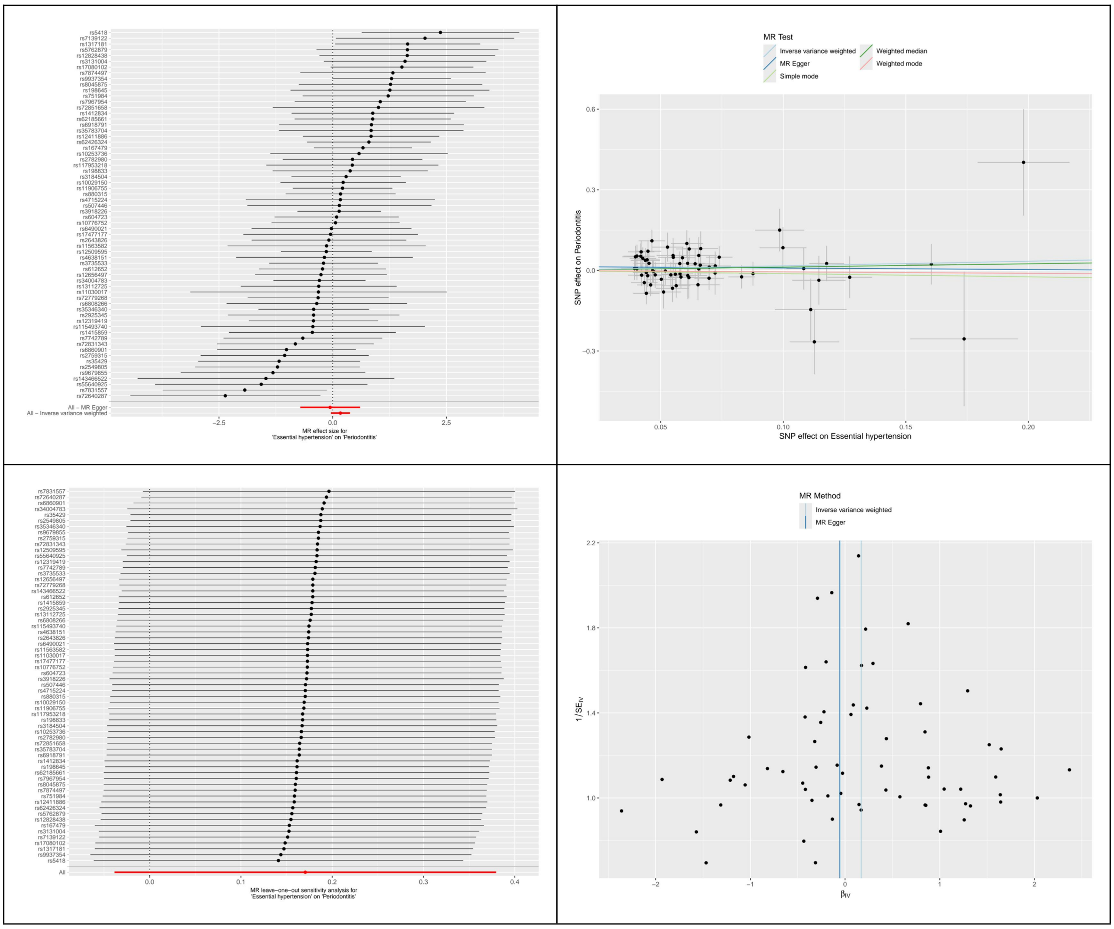

Essential hypertension on Periodontitis

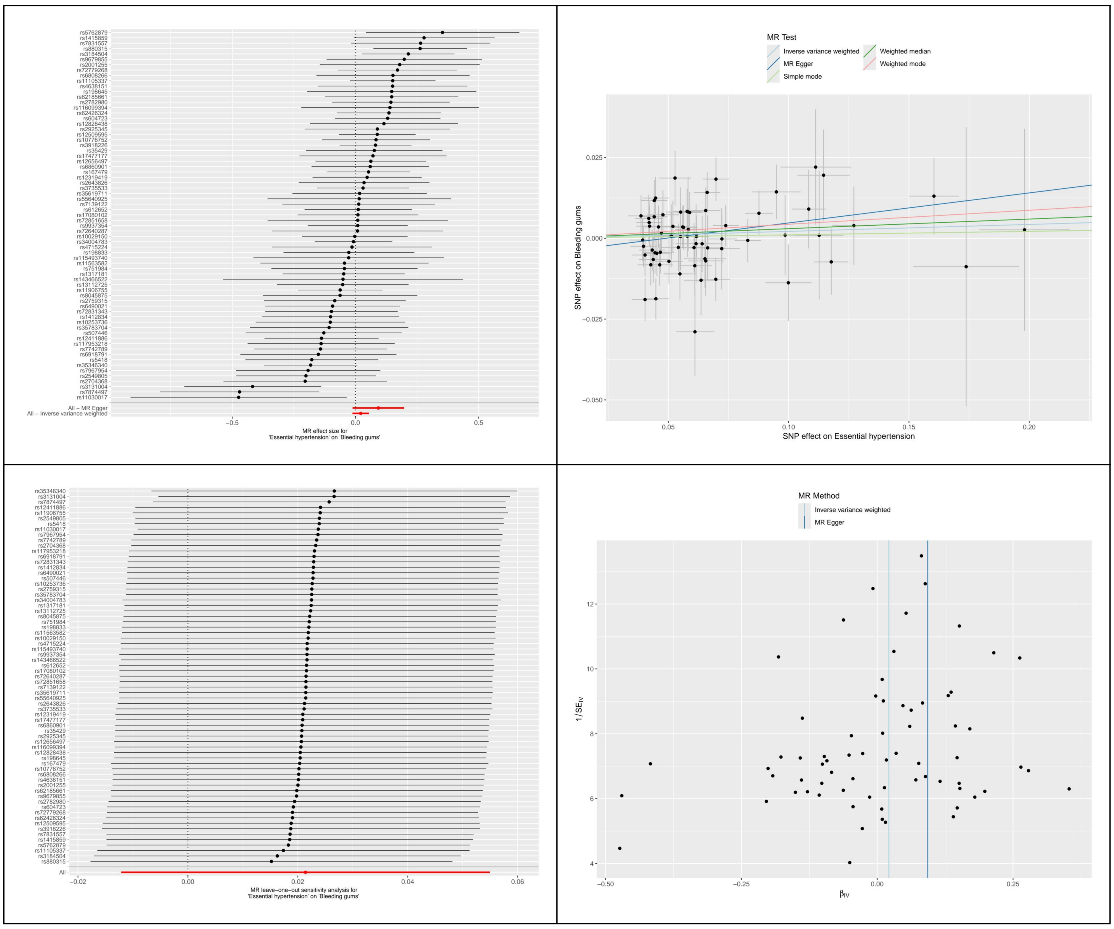

Essential hypertension on Bleeding gums

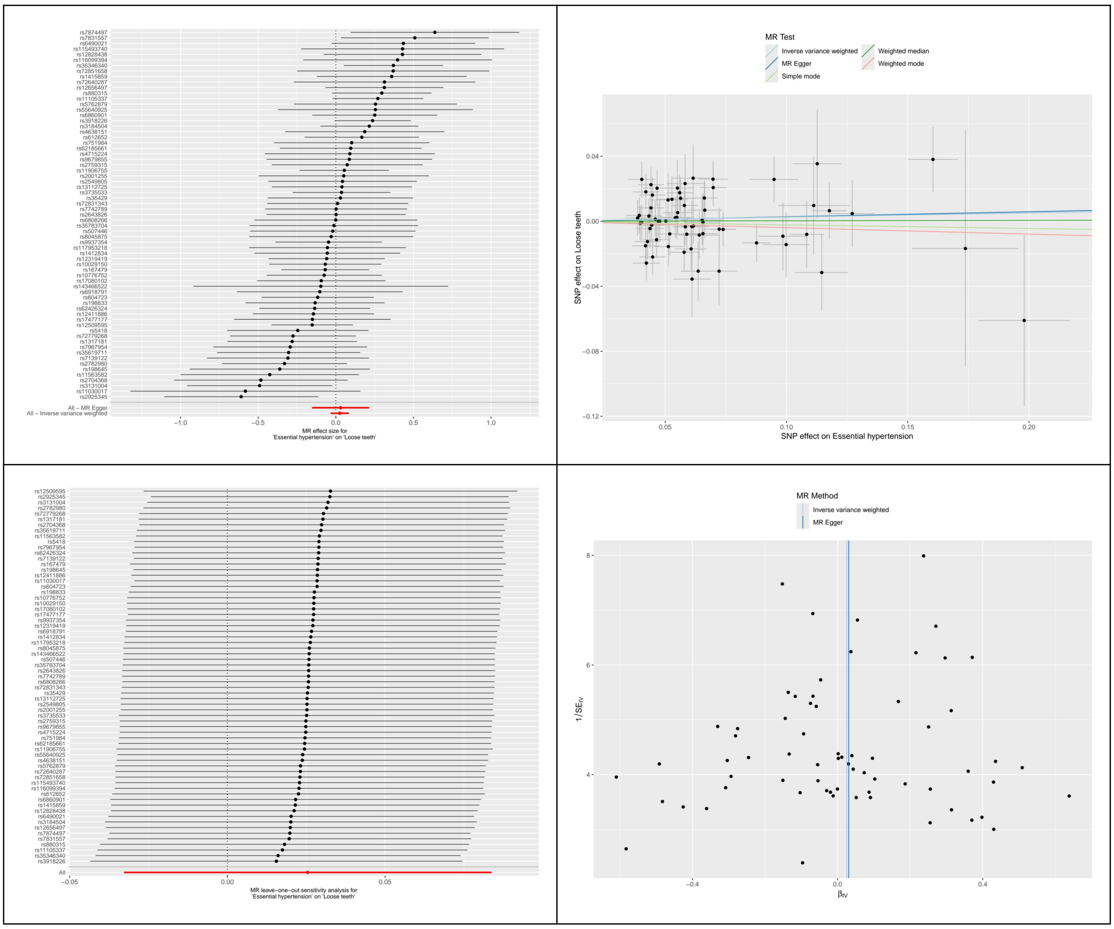

Essential hypertension on Loose teeth

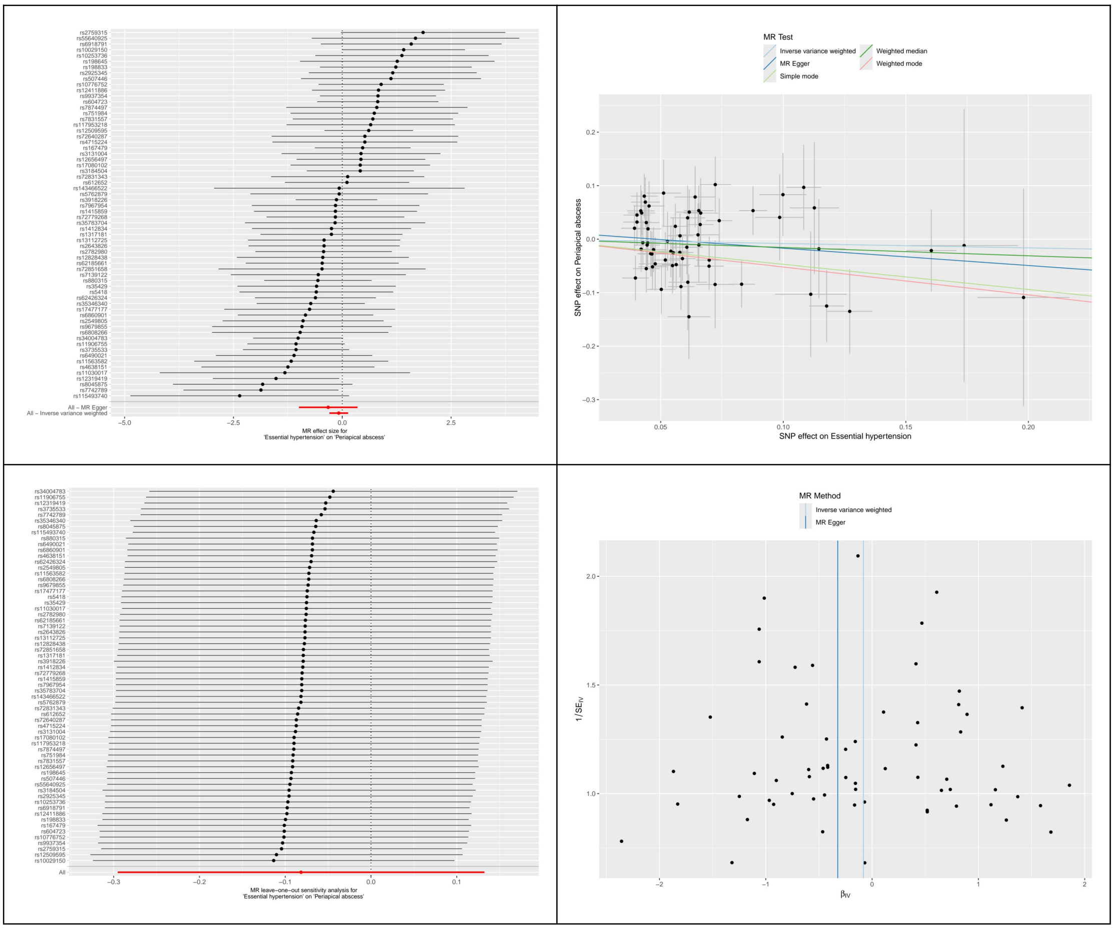

Essential hypertension on Periapical abscess

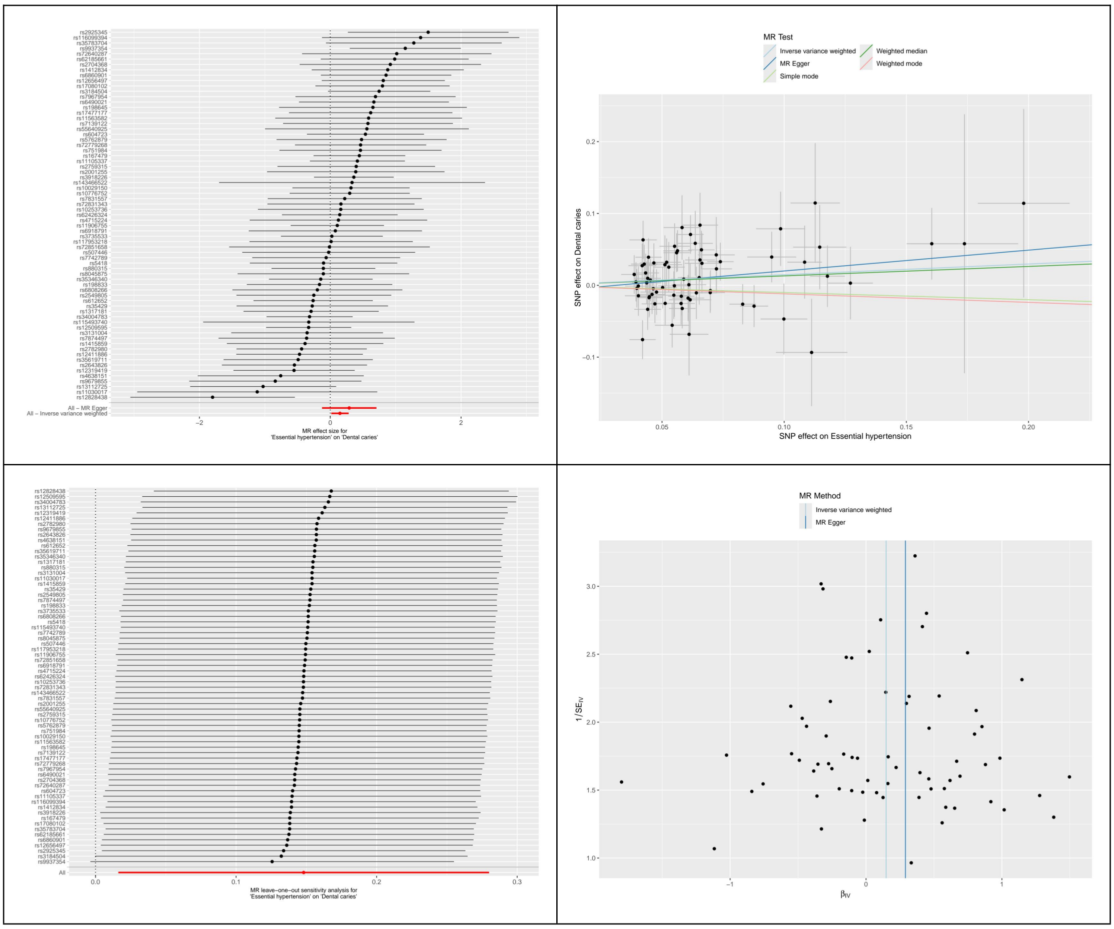

Essential hypertension on Dental caries

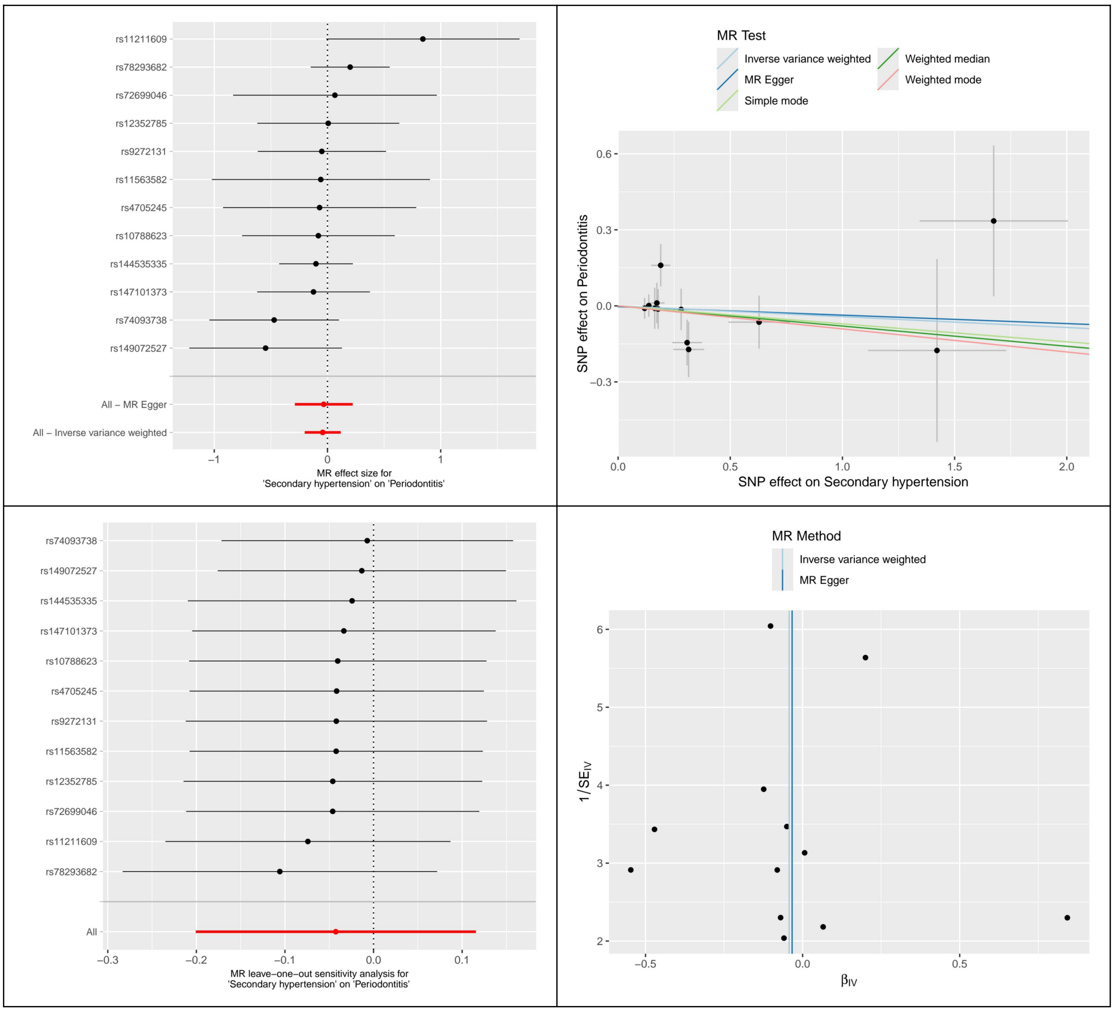

Secondary hypertension on Periodontitis

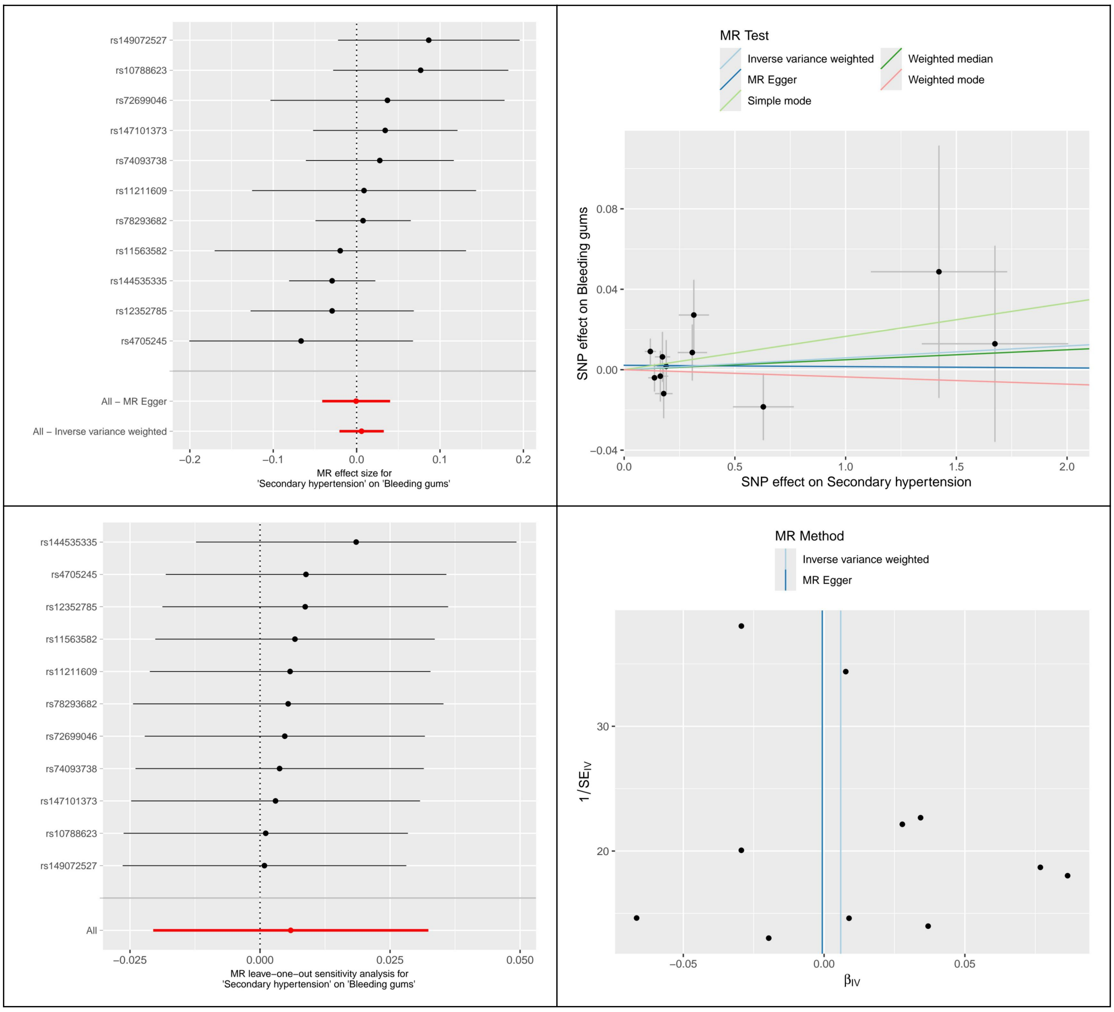

Secondary hypertension on Bleeding gums

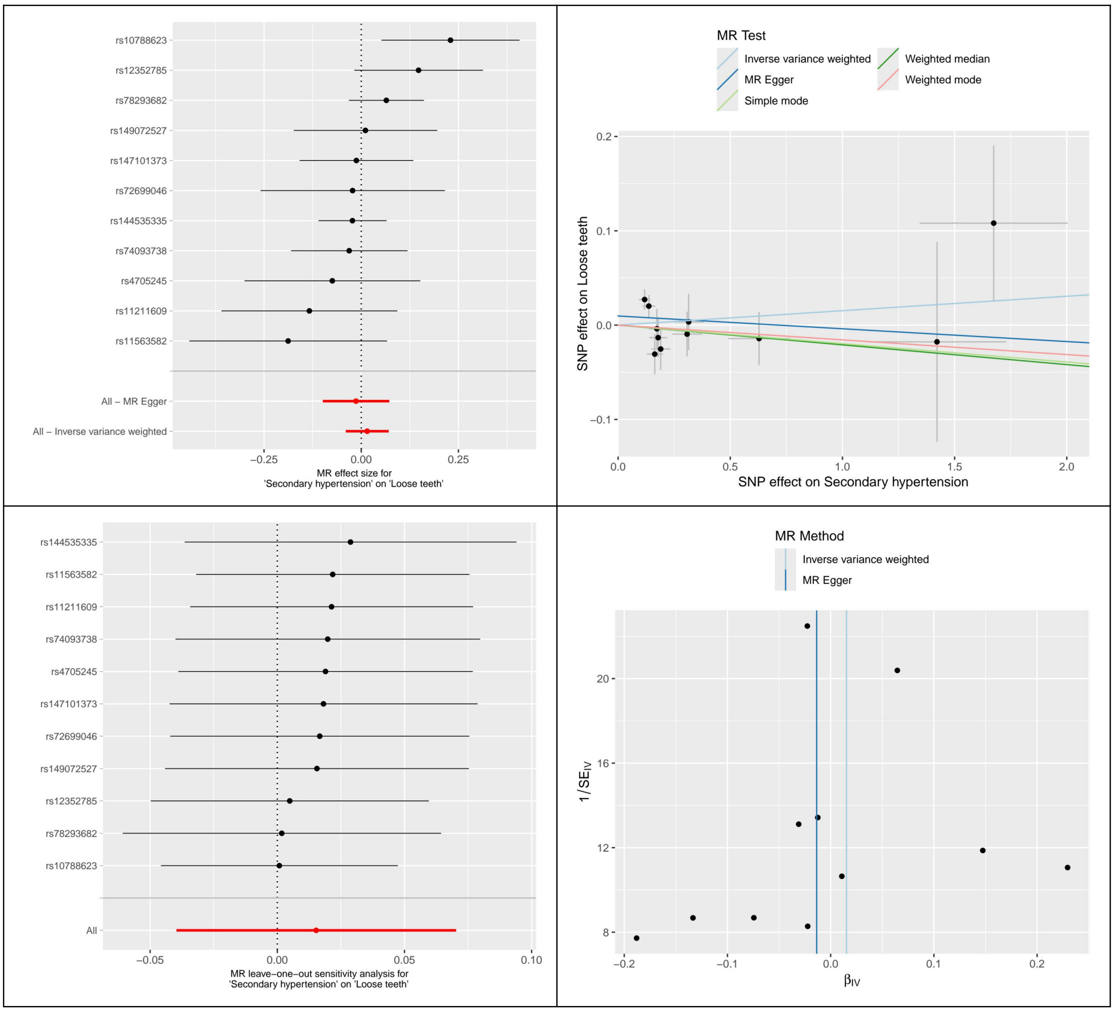

Secondary hypertension on Loose teeth

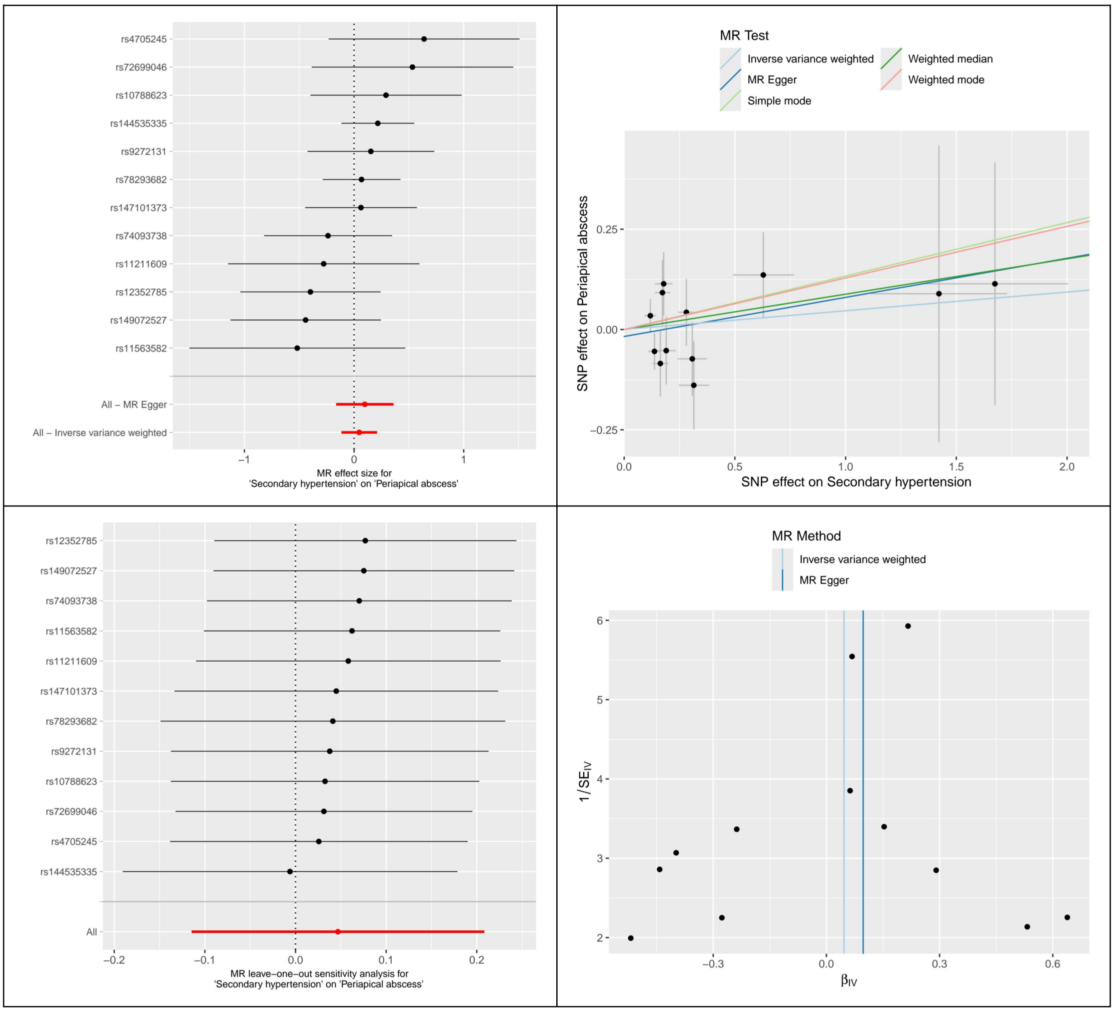

Secondary hypertension on Periapical abscess

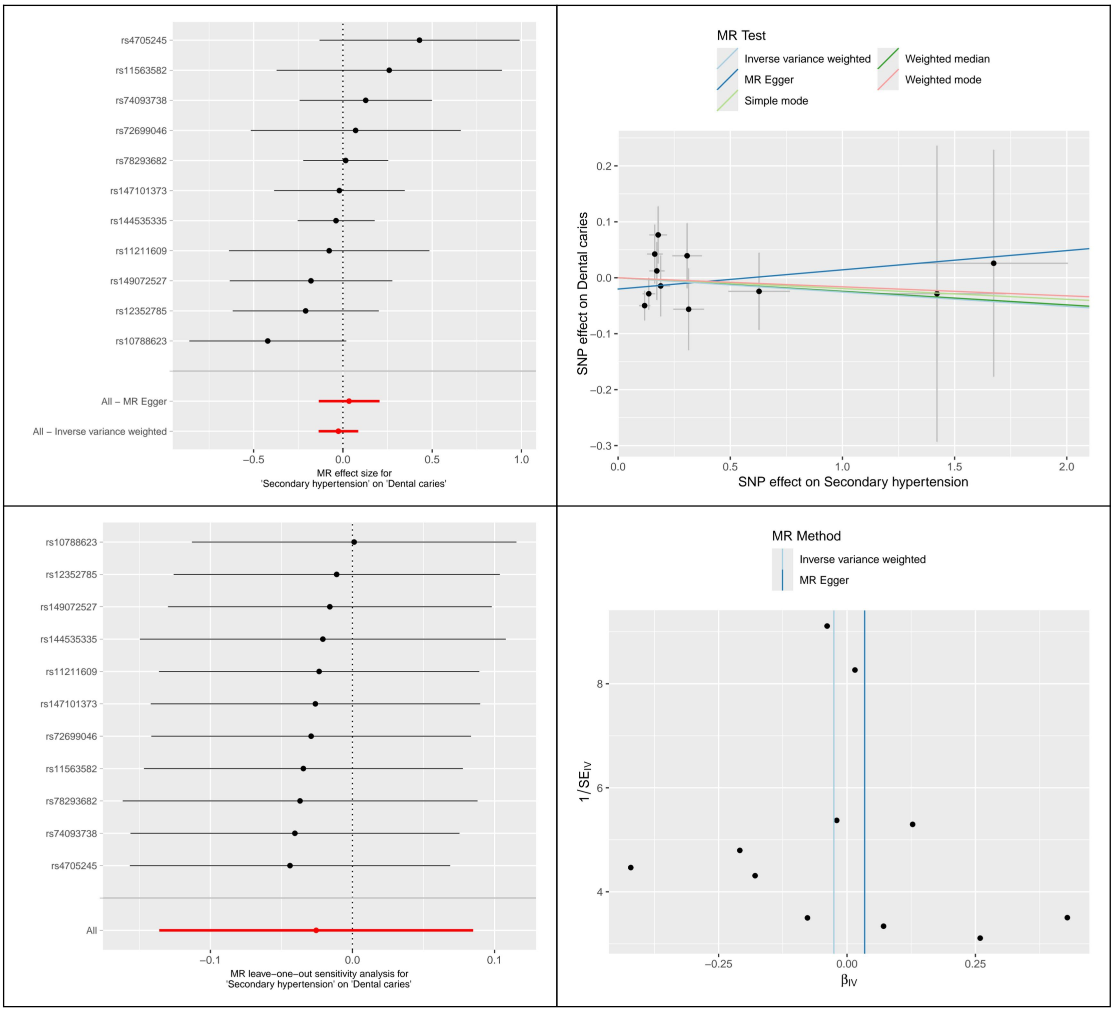

Secondary hypertension on Dental caries
