# Supplementary material for: Causal relationship between oral diseases and hypertension: a Mendelian randomization study
Source: Exp Biol Med (Maywood). 2026 May 7;251:10922. doi: 10.3389/ebm.2026.10922 (PMC13189983; doi:10.3389/ebm.2026.10922)
Supplement: Supplementary file 2 [file DataSheet3.pdf]

Results of Steiger test

Exposure	Outcome	r^2.exposure	r^2.outcome	Correct causal direction	Steiger *P*
Periodontitis	Essential hypertension	3.44E-04	1.84E-05	TRUE	<0.05
Bleeding gums		6.84E-04	4.68E-05	TRUE	<0.05
Loose teeth		7.81E-04	4.01E-05	TRUE	<0.05
Periapical abscess		4.93E-04	2.93E-05	TRUE	<0.05
Dental caries		5.06E-04	3.75E-05	TRUE	<0.05
Essential hypertension	Periodontitis	1.36E-02	1.92E-04	TRUE	<0.05
	Bleeding gums	1.43E-02	2.05E-04	TRUE	<0.05
	Loose teeth	1.37E-02	1.95E-04	TRUE	<0.05
	Periapical abscess	1.36E-02	1.87E-04	TRUE	<0.05
	Dental caries	1.43E-02	1.85E-04	TRUE	<0.05
Secondary hypertension	Periodontitis	1.28E-03	2.99E-05	TRUE	<0.05
	Bleeding gums	1.05E-03	2.66E-05	TRUE	<0.05
	Loose teeth	1.05E-03	3.48E-05	TRUE	<0.05
	Periapical abscess	1.28E-03	3.11E-05	TRUE	<0.05
	Dental caries	1.05E-03	2.65E-05	TRUE	<0.05
