# Supplementary material for: Causal relationship between oral diseases and hypertension: a Mendelian randomization study
Source: Exp Biol Med (Maywood). 2026 May 7;251:10922. doi: 10.3389/ebm.2026.10922 (PMC13189983; doi:10.3389/ebm.2026.10922)
Supplement: Supplementary file 3 [file DataSheet1.pdf]

Instrumental variables used in the MR analysis

Trait	SNP	Effect allele	Other allele	Beta	EAF	*P* value	SE	*F*
Periodontitis	rs7518774	T	C	0.663	0.023086	2.78E-06	0.141	22.11
Periodontitis	rs13387840	A	G	0.25	0.184375	1.81E-06	0.0524	22.76237
Periodontitis	rs10006765	G	C	0.862	0.016965	2.56E-06	0.183	22.1877
Periodontitis	rs11167673	C	G	-0.221	0.756678	3.31E-06	0.0476	21.55612
Periodontitis	rs62519766	A	G	0.484	0.049338	5.69E-07	0.0969	24.94843
Periodontitis	rs11602337	T	C	-0.211	0.297646	2.24E-06	0.0446	22.38181
Periodontitis	rs8069681	T	A	0.21	0.319898	1.40E-06	0.0435	23.30559
Bleeding gums	rs12123214	T	C	0.04906	0.147207	4.65E-08	0.008978	29.8573
Bleeding gums	rs149037306	C	T	-0.06611	0.065018	3.62E-07	0.012994	25.8845
Bleeding gums	rs11717033	C	T	0.083571	0.039746	3.09E-07	0.016329	26.19181
Bleeding gums	rs10948120	A	G	0.041348	0.26606	1.62E-08	0.00732	31.90357
Bleeding gums	rs4946822	A	T	0.043939	0.171288	2.01E-07	0.008453	27.01901
Bleeding gums	rs59489169	A	C	-0.0324	0.485463	3.09E-07	0.00633	26.19438
Bleeding gums	rs17151682	G	T	0.036817	0.27992	1.62E-07	0.007029	27.4373
Bleeding gums	rs7125773	T	C	-0.03777	0.303502	3.75E-08	0.006865	30.27612
Bleeding gums	rs4570722	C	T	0.037896	0.492603	1.88E-09	0.006307	36.09774
Bleeding gums	rs150064030	A	G	0.067879	0.067843	8.10E-08	0.012653	28.78094
Bleeding gums	rs73313647	T	A	-0.038	0.236274	3.57E-07	0.007466	25.91137
Bleeding gums	rs111692872	T	G	-0.08025	0.041399	4.89E-07	0.015954	25.30581
Bleeding gums	rs117686655	A	G	0.114127	0.020272	3.93E-07	0.0225	25.72857
Bleeding gums	rs6025146	G	A	-0.0399	0.248984	4.95E-08	0.007318	29.73501
Loose teeth	rs115723382	A	C	0.305622	0.006788	3.84E-06	0.066156	21.34172
Loose teeth	rs41266241	C	G	-0.11494	0.063158	1.48E-07	0.021874	27.61321
Loose teeth	rs181728410	C	G	-0.05338	0.301412	4.84E-06	0.011676	20.90021
Loose teeth	rs72664596	A	C	0.102443	0.064296	2.64E-06	0.021811	22.06
Loose teeth	rs77239350	T	G	0.169199	0.022274	2.88E-06	0.03616	21.89529
Loose teeth	rs137904400	A	G	0.290523	0.008289	1.06E-06	0.059539	23.81016
Loose teeth	rs3763469	C	T	-0.06151	0.218053	2.76E-06	0.013121	21.97555
Loose teeth	rs11220245	G	A	0.061839	0.246964	5.93E-07	0.012384	24.93464
Loose teeth	rs10746307	A	C	-0.06339	0.237088	4.30E-07	0.01254	25.55616
Loose teeth	rs2768202	G	T	-0.21968	0.013411	2.56E-06	0.046706	22.12158
Loose teeth	rs982894	A	G	-0.05373	0.363765	1.23E-06	0.011078	23.52747
Loose teeth	rs114873975	A	G	0.332401	0.007538	1.29E-07	0.062943	27.88844
Loose teeth	rs12903285	A	G	-0.05159	0.388062	2.69E-06	0.010993	22.02492
Loose teeth	rs312989	G	A	0.055131	0.360099	6.52E-07	0.011081	24.75155
Loose teeth	rs117295134	A	G	0.128661	0.038269	3.67E-06	0.027794	21.42927
Loose teeth	rs714962	G	A	0.053268	0.314961	3.73E-06	0.011515	21.39985
Periapical abscess	rs185710552	A	G	1.19	0.008422	4.57E-06	0.259	21.1103

Periapical abscess	rs11690772	G	C	0.468	0.048715	3.40E-06	0.101	21.47084
Periapical abscess	rs115567550	A	G	0.967	0.013602	1.22E-06	0.199	23.61276
Periapical abscess	rs537018853	C	T	1.14	0.010451	2.25E-06	0.242	22.19111
Periapical abscess	rs17619647	T	C	0.199	0.420454	2.40E-06	0.0423	22.13224
Periapical abscess	rs34016503	G	A	0.496	0.043928	3.06E-06	0.106	21.89534
Periapical abscess	rs141402469	G	A	1.26	0.008941	2.71E-06	0.269	21.94
Periapical abscess	rs7099542	G	A	-1.2	0.992279	4.64E-06	0.262	20.9778
Periapical abscess	rs11819509	A	G	0.434	0.053953	4.24E-06	0.0944	21.13662
Dental caries	rs11678150	A	T	0.125699	0.391299	3.32E-06	0.027032	21.62305
Dental caries	rs10939619	A	G	0.124843	0.403683	3.72E-06	0.026986	21.40169
Dental caries	rs77030061	T	C	0.332285	0.039291	1.54E-06	0.069136	23.10012
Dental caries	rs147891457	A	G	0.727966	0.009546	5.05E-07	0.144881	25.24639
Dental caries	rs150690575	C	T	0.90055	0.006166	9.33E-07	0.183588	24.06174
Dental caries	rs4707035	A	C	-0.14904	0.245887	1.97E-06	0.031331	22.62841
Dental caries	rs41272372	T	C	0.222809	0.091529	4.44E-06	0.048545	21.06573
Dental caries	rs116960995	T	C	0.501808	0.01758	1.45E-06	0.104161	23.20945
Dental caries	rs2942688	T	C	-0.19578	0.115393	2.27E-06	0.041414	22.34942
Dental caries	rs10420876	A	G	0.130187	0.343037	4.32E-06	0.02833	21.11688
Essential hypertension	rs880315	C	T	0.069807	0.412185	1.47E-38	0.005376	168.6371
Essential hypertension	rs72640287	T	C	-0.11269	0.077514	3.02E-29	0.010038	126.0388
Essential hypertension	rs4638151	T	C	-0.04412	0.628165	7.09E-16	0.005468	65.10759
Essential hypertension	rs10776752	T	G	0.108359	0.170282	7.66E-54	0.007014	238.6724
Essential hypertension	rs1317181	T	G	0.060672	0.209256	9.78E-21	0.006497	87.20493
Essential hypertension	rs34004783	G	C	-0.08301	0.594011	1.35E-53	0.005386	237.545
Essential hypertension	rs116099394	T	G	-0.0582	0.145963	8.70E-15	0.007504	60.16936
Essential hypertension	rs2704368	G	A	-0.0636	0.836954	6.63E-19	0.007162	78.87111
Essential hypertension	rs2643826	T	C	0.047095	0.403423	2.43E-18	0.005391	76.30261
Essential hypertension	rs6808266	G	A	0.047765	0.798786	5.02E-13	0.006611	52.19773
Essential hypertension	rs12509595	C	T	0.087669	0.31293	3.08E-53	0.005708	235.8994
Essential hypertension	rs13112725	C	G	0.054156	0.828359	1.55E-14	0.007048	59.03442
Essential hypertension	rs2001255	T	C	-0.03863	0.495102	3.19E-13	0.005301	53.08972
Essential hypertension	rs10029150	G	C	0.072269	0.774443	3.65E-30	0.006333	130.2279
Essential hypertension	rs12656497	C	T	0.055981	0.58523	1.58E-25	0.005361	109.0545
Essential hypertension	rs188155432	T	C	0.138483	0.023605	1.59E-15	0.017376	63.51603
Essential hypertension	rs2549805	A	T	0.054827	0.807665	4.23E-16	0.006742	66.1247
Essential hypertension	rs6860901	T	C	0.056336	0.297226	1.65E-22	0.005771	95.28704
Essential hypertension	rs4371736	C	G	-0.04702	0.382255	6.05E-18	0.005447	74.5028
Essential hypertension	rs198833	A	G	-0.06405	0.889362	2.20E-14	0.008386	58.34661
Essential hypertension	rs3131004	G	A	0.044826	0.450254	3.66E-17	0.005322	70.95319
Essential hypertension	rs115493740	A	G	0.061521	0.104807	9.41E-13	0.008618	50.96326
Essential hypertension	rs7742789	T	C	0.050253	0.294155	3.43E-18	0.005779	75.62686
Essential hypertension	rs4715224	A	T	0.039335	0.397773	3.56E-13	0.00541	52.87269

Essential hypertension	rs1415859	G	A	0.044722	0.410039	9.09E-17	0.005378	69.15626
Essential hypertension	rs6918791	G	C	0.043738	0.728148	2.11E-13	0.005957	53.90386
Essential hypertension	rs62426324	T	C	0.058884	0.500267	8.65E-29	0.005289	123.9484
Essential hypertension	rs198645	T	A	-0.04189	0.332313	8.98E-14	0.005619	55.5779
Essential hypertension	rs17080102	C	G	-0.09854	0.076127	7.08E-23	0.010007	96.95801
Essential hypertension	rs3735533	C	T	0.127106	0.904426	4.65E-44	0.00913	193.827
Essential hypertension	rs11563582	A	G	0.072203	0.146367	8.16E-22	0.007523	92.11897
Essential hypertension	rs17477177	C	T	0.051902	0.297918	2.60E-19	0.005777	80.7214
Essential hypertension	rs3918226	T	C	0.160397	0.069854	6.44E-55	0.010277	243.6044
Essential hypertension	rs10253736	T	C	0.04525	0.231017	6.33E-13	0.006291	51.74087
Essential hypertension	rs7831557	A	G	-0.04414	0.466821	1.29E-16	0.005335	68.46017
Essential hypertension	rs35783704	A	G	-0.06556	0.155344	4.34E-19	0.007343	79.70613
Essential hypertension	rs1412834	C	T	0.04447	0.433834	7.37E-17	0.005331	69.57205
Essential hypertension	rs7874497	G	A	0.040331	0.627505	2.35E-13	0.005504	53.68644
Essential hypertension	rs72779268	A	C	-0.11454	0.064498	5.21E-26	0.010859	111.2526
Essential hypertension	rs188460025	T	C	-0.07431	0.069974	1.13E-12	0.010445	50.60991
Essential hypertension	rs72831343	G	T	-0.06523	0.102354	9.17E-14	0.008753	55.53681
Essential hypertension	rs2274224	C	G	-0.04402	0.344749	3.04E-15	0.00558	62.24044
Essential hypertension	rs12411886	A	C	-0.09988	0.085072	1.12E-25	0.009535	109.7384
Essential hypertension	rs1265002	T	A	-0.09153	0.952544	1.71E-13	0.012421	54.3079
Essential hypertension	rs2782980	C	T	0.057809	0.653565	2.73E-25	0.005563	107.968
Essential hypertension	rs612652	C	T	0.057898	0.573315	2.34E-27	0.005343	117.4091
Essential hypertension	rs55640925	A	C	-0.05119	0.17512	3.51E-13	0.007038	52.9004
Essential hypertension	rs72851658	A	T	0.055064	0.196663	1.65E-16	0.006678	67.98447
Essential hypertension	rs11030017	T	C	-0.06107	0.871443	8.32E-15	0.007867	60.25808
Essential hypertension	rs751984	C	T	-0.06634	0.171177	5.42E-21	0.007057	88.3737
Essential hypertension	rs604723	C	T	0.065549	0.740301	1.98E-27	0.006041	117.7327
Essential hypertension	rs12828438	G	A	-0.04203	0.53466	2.85E-15	0.005322	62.36815
Essential hypertension	rs7967954	A	G	-0.04272	0.589981	1.63E-15	0.005362	63.4692
Essential hypertension	rs7139122	A	G	0.198021	0.020175	3.75E-26	0.018719	111.9022
Essential hypertension	rs12319419	C	G	-0.05827	0.408084	2.47E-27	0.005381	117.2989
Essential hypertension	rs11105337	T	A	-0.09489	0.077206	1.66E-21	0.009963	90.71524
Essential hypertension	rs3184504	C	T	-0.06613	0.593438	9.77E-35	0.005379	151.1384
Essential hypertension	rs35429	G	A	-0.04592	0.364897	7.01E-17	0.005502	69.67107
Essential hypertension	rs6490021	C	A	0.046666	0.695978	5.77E-16	0.005765	65.51388
Essential hypertension	rs507446	T	C	0.04032	0.578739	6.38E-14	0.005376	56.25077
Essential hypertension	rs2925345	C	T	-0.04224	0.648966	2.61E-14	0.005546	58.01081
Essential hypertension	rs2759315	A	C	0.043304	0.400332	1.07E-15	0.0054	64.30012
Essential hypertension	rs35346340	C	G	0.069737	0.270582	1.13E-31	0.005955	137.1234
Essential hypertension	rs9937354	A	G	0.061658	0.427884	6.99E-31	0.005336	133.5112
Essential hypertension	rs8045875	C	T	0.039694	0.59387	1.75E-13	0.005388	54.27282
Essential hypertension	rs5418	A	G	0.046426	0.602341	8.58E-18	0.005404	73.81582
Essential hypertension	rs117953218	C	T	0.060995	0.169745	4.33E-18	0.007035	75.16325
Essential hypertension	rs35619711	G	C	0.051297	0.7207	3.42E-18	0.005899	75.62846

Essential hypertension	rs143466522	A	G	0.173777	0.014603	2.27E-15	0.021926	62.81707
Essential hypertension	rs167479	G	T	0.073877	0.575821	4.54E-43	0.00537	189.2904
Essential hypertension	rs62185661	A	G	0.055148	0.242315	4.51E-19	0.00618	79.63353
Essential hypertension	rs11906755	G	A	0.117696	0.163536	1.77E-61	0.007114	273.7001
Essential hypertension	rs9679855	G	A	0.111216	0.033403	2.57E-14	0.014599	58.03875
Essential hypertension	rs2427541	G	C	-0.03818	0.501283	6.56E-13	0.005311	51.67198
Essential hypertension	rs5762879	T	C	0.052776	0.212159	3.10E-16	0.00646	66.7407
Secondary hypertension	rs11211609	C	T	0.1899	0.091838	4.74E-06	0.041499	20.9398
Secondary hypertension	rs72699046	C	G	0.172812	0.154021	2.67E-07	0.033586	26.47503
Secondary hypertension	rs78293682	T	C	-1.67423	0.003931	3.89E-07	0.329931	25.7504
Secondary hypertension	rs13100724	A	T	0.114925	0.53644	4.38E-06	0.025025	21.0897
Secondary hypertension	rs4705245	C	T	0.178532	0.87268	4.70E-06	0.039001	20.95434
Secondary hypertension	rs144535335	T	C	-0.62837	0.011921	4.37E-06	0.136809	21.096
Secondary hypertension	rs9272131	T	C	0.280992	0.111358	3.51E-14	0.03708	57.42537
Secondary hypertension	rs11563582	A	G	0.163401	0.145569	2.89E-06	0.034925	21.88904
Secondary hypertension	rs147101373	T	G	1.42144	0.000788	3.69E-06	0.307119	21.4212
Secondary hypertension	rs12352785	C	A	-0.13689	0.747412	9.81E-07	0.027962	23.96506
Secondary hypertension	rs10788623	A	G	-0.11822	0.511552	1.97E-06	0.024858	22.6202
Secondary hypertension	rs149072527	T	C	0.314548	0.029084	3.52E-06	0.067818	21.51234
Secondary hypertension	rs74093738	A	G	-0.30762	0.04443	2.90E-06	0.065759	21.88283
